# Autonomy-Supportive Healthcare, Medication Adherence, and Life Satisfaction in Adolescents with Epilepsy: A Cross-Sectional Association

**DOI:** 10.3390/bs16091569

**Published:** 2026-09-04

**Authors:** Valeria Verrastro, Valeria Saladino, Andrea Sgroi, Danilo Calaresi

**Affiliations:** 1 Department of Health Sciences, Magna Graecia University, 88100 Catanzaro, Italy; valeriaverrastro@unicz.it (V.V.); v.saladino@unicz.it (V.S.); 2 Department of Humanistic Research and Innovation, University of Bari Aldo Moro, 70121 Bari, Italy; a.sgroi1@phd.uniba.it; 3 Faculty of Communication, Culture and Society, University of Italian Switzerland, 6900 Lugano, Switzerland

**Keywords:** adolescents, epilepsy, medication adherence, life satisfaction, Self-Determination Theory

## Abstract

Adolescence is a critical developmental period in which epilepsy can affect both health behaviors and psychological well-being. However, clinical indicators alone do not fully capture adolescents’ lived experience. Grounded in Self-Determination Theory, this study examined the cross-sectional associations among autonomy-supportive healthcare (ASH) environments, medication adherence, and life satisfaction in adolescents with epilepsy. A cross-sectional design was used with 1219 Italian adolescents with epilepsy (*M* age = 15.58, *SD* = 1.72), recruited through pediatric neurology clinics and national patient support organizations. Participants completed validated self-report measures: the Health Care Climate Questionnaire (HCCQ) for ASH, the Medication Adherence Report Scale (MARS) for medication adherence, and the Satisfaction With Life Scale (SWLS) for life satisfaction. A mediation model was tested to evaluate the indirectly associated paths among the variables. The mediation model showed a good fit to the data (χ^2^/df = 2.45, CFI = 0.994, RMSEA = 0.034, SRMR = 0.029). ASH was positively associated with both medication adherence (*β* = 0.40, *p* < 0.001) and life satisfaction (*β* = 0.23, *p* < 0.001), while medication adherence was positively associated with life satisfaction (*β* = 0.35, *p* < 0.001). The indirect statistical path from ASH to life satisfaction through medication adherence was also significant (*β* = 0.08, *p* < 0.001), indicating partial mediation. Overall, these findings highlight positive cross-sectional links between ASH environments, medication adherence, and psychological well-being in adolescents with epilepsy, underscoring the potential relevance of relational aspects of care in chronic disease management.

## 1. Introduction

Adolescence is a critical developmental period characterized by rapid biological, psychological, and social changes that jointly shape trajectories of health and well-being (Giordano et al., 2025; Silvers, 2022; Verrastro et al., 2025). Neurodevelopmental maturation, particularly within prefrontal and limbic systems, supports increasing cognitive control and social functioning, while also heightening emotional reactivity and sensitivity to social context (Haft et al., 2021; Li et al., 2025). Concurrently, youth face key developmental tasks related to identity formation, autonomy striving, and expanding social relationships, which occur within increasingly complex peer, school, and digital environments (Branje et al., 2021; Calaresi et al., 2024; Saladino et al., 2024). These normative developmental processes become especially relevant in the context of a chronic illness, such as epilepsy. Epilepsy is a neurological condition characterized by recurrent, often unpredictable seizures that can affect cognitive functioning, emotional regulation, and social participation (Karoly et al., 2021). Beyond its medical characteristics, epilepsy can interfere with adolescents’ autonomy development, school participation, and peer relationships, and may also expose individuals to stigma or social restriction (Hyland et al., 2025; Yang et al., 2022). Given these multidimensional challenges, outcomes in adolescent epilepsy cannot be fully understood through clinical indicators alone. Increasing attention has therefore been placed on subjective well-being, particularly life satisfaction, defined as individuals’ global cognitive evaluation of their quality of life (Aymerich et al., 2021; Daly, 2022). Life satisfaction is a key indicator of psychosocial adjustment during adolescence, reflecting whether individuals perceive their lives as meaningful and satisfying across domains such as family, peers, school, and future orientation. However, life satisfaction in chronic illness contexts is shaped by both behavioral and contextual processes. Two factors grounded in motivational and health behavior processes are particularly relevant: (1) medication adherence, defined as the extent to which individuals follow prescribed treatment regimens (Kvarnström et al., 2021), and (2) autonomy-supportive healthcare (ASH), defined as the extent to which healthcare providers support patients’ autonomy through empathy, acknowledgment of their perspectives, and involvement in treatment-related decisions (Upadhyai et al., 2019). Medication adherence is essential for seizure control and long-term health stability and is influenced by cognitive, emotional, and social factors, particularly during adolescence. ASH, in turn, represents a key contextual determinant of how adolescents engage with treatment and adapt psychologically to chronic illness. Together, these factors may jointly shape adolescents’ behavioral engagement with treatment and their subjective well-being.

### 1.1. Self-Determination Theory as a Theoretical Framework

Self-Determination Theory (SDT) provides a theoretical foundation for understanding how healthcare contexts influence motivation, health behavior, and well-being (Ryan & Deci, 2023). SDT posits that social environments shape motivation through the extent to which they support individuals’ basic psychological needs for autonomy, competence, and relatedness, thereby facilitating the internalization of health-related behaviors. Within this framework, motivation is conceptualized along a continuum ranging from controlled forms, driven by external pressure or obligation, to more autonomous forms, in which behaviors are self-endorsed and integrated into the self. When healthcare contexts support psychological needs, individuals are more likely to internalize treatment recommendations and engage in health behaviors in a more self-determined manner; when needs are undermined, adherence tends to be more externally regulated and less stable over time. In healthcare settings, ASH represents a key contextual expression of SDT principles. ASH is characterized by empathic communication, acknowledgment of patients’ perspectives, and involvement in treatment-related decisions. Such environments are expected to foster more autonomous regulation of treatment behaviors, thereby promoting more consistent engagement with medical recommendations. In contrast, controlling healthcare styles, which emphasize compliance without patient involvement, may undermine perceived autonomy and weaken sustained adherence. During adolescence, these processes may be particularly relevant, as this developmental period is marked by increased sensitivity to autonomy-related experiences and heightened need for self-endorsement in health-related decision-making. As such, SDT offers a useful framework for understanding how the quality of healthcare interactions may influence adolescents’ adherence to treatment and, ultimately, their psychological well-being.

### 1.2. Empirical Grounding of the Self-Determination Theory Model

A growing body of research suggests that adolescents’ adjustment to chronic illness is shaped by a dynamic interplay between contextual factors, health behaviors, and psychological well-being. Within this framework, evidence consistently indicates that the quality of the healthcare environment is associated with adolescents’ engagement in treatment behaviors, particularly medication adherence. ASH, characterized by empathic communication, acknowledgment of patients’ perspectives, and involvement in treatment-related decisions, has been linked to greater treatment engagement and self-management in chronic illness populations (Barello et al., 2020; Conn et al., 2015). In contrast, controlling or directive clinical environments are generally associated with lower adherence, likely due to reduced perceived autonomy and weaker internalization of treatment recommendations (Allen-Duck et al., 2017; Koster et al., 2015). Overall, these findings suggest that healthcare context plays a relevant role in shaping adolescents’ behavioral engagement with medical treatment.

Beyond adherence, ASH has also been associated with psychological well-being. Autonomy-supportive interpersonal contexts have been linked to higher life satisfaction and better psychosocial functioning, as they support more positive and self-endorsed experiences of care (Chen et al., 2022; Goel et al., 2018; Jordan et al., 2018). In adolescents with chronic conditions, these processes may be particularly relevant given the simultaneous demands of treatment management, identity development, and increasing autonomy. Medication adherence has itself been associated with psychological outcomes, including life satisfaction. In epilepsy, better adherence is linked to improved seizure control, greater stability, and reduced uncertainty, which contribute to a more positive evaluation of daily life (Byansi et al., 2023; Ismail et al., 2024). Conversely, non-adherence may increase health instability and emotional burden, limiting adolescents’ participation in everyday activities (Kretchy et al., 2020; Saedi et al., 2024). Grounded in SDT, medication adherence is hypothesized as a crucial behavioral mechanism translating autonomy-supportive care into broader subjective well-being (Ryan & Deci, 2023). When healthcare providers adopt an autonomy-supportive style, acknowledging perspectives and supporting self-initiation, adolescents internalize the value of their treatment rather than viewing it as an external imposition, fostering self-regulated behavioral adherence (Allen-Duck et al., 2017). In the context of adolescent epilepsy, consistent adherence is not merely a clinical end point; it directly minimizes unpredictable seizure recurrences, medical complications, and lifestyle restrictions (Karoly et al., 2021). By gaining behavioral control over their condition, adolescents experience reduced health anxiety and greater freedom to participate in normative peer, academic, and social activities (Hyland et al., 2025). Thus, medication adherence serves as a key behavioral pathway through which a supportive healthcare climate translates into higher perceived quality of life and global life satisfaction (Aymerich et al., 2021).

### 1.3. Aims and Hypotheses

Despite growing recognition of the role of autonomy support in pediatric chronic care, significant gaps remain in the literature. First, while SDT principles have been applied broadly across adult and general chronic illness management, empirical investigations specifically examining ASH in adolescent epilepsy remain limited. Adolescence in epilepsy presents a unique intersection of developmental demands for autonomy and the intrusive, unpredictable nature of seizure management. Second, although individual links between ASH, medication adherence, and subjective well-being are established, few studies have tested an integrated indirect model evaluating whether medication adherence serves as a behavioral mechanism linking healthcare context to global life satisfaction. Addressing these gaps is crucial for clarifying how clinical communication styles translate into both behavioral engagement and broader psychological adjustment in youth with epilepsy.

This cross-sectional study examined the associations among ASH, medication adherence, and life satisfaction in adolescents with epilepsy. Building on prior evidence highlighting the relevance of healthcare context and treatment engagement for psychological adjustment in chronic illness, the study addressed three main objectives: (a) to examine the association between ASH and medication adherence; (b) to examine the association between ASH and life satisfaction; and (c) to assess the association between medication adherence and life satisfaction. Finally, (d) we explored whether medication adherence mediates the relationship between ASH and life satisfaction. We hypothesized that ASH would be positively associated with both medication adherence and life satisfaction, and that medication adherence would also be positively associated with life satisfaction. Finally, medication adherence was expected to act as a potential mediator in the association between ASH and life satisfaction.

## 2. Methods

### 2.1. Participants

The study included 1219 adolescents with a clinical diagnosis of epilepsy, aged between 13 and 18 years (*M* = 15.58, *SD* = 1.72). Participants were recruited through a multi-source strategy aimed at increasing heterogeneity and external validity. Specifically, participants were recruited between October 2024 and January 2025 across several recruitment sites included neurology clinics, outpatient services, and hospitals specializing in epilepsy care, as well as epilepsy associations and community outreach programs located in Northern, Central, and Southern Italy. This approach allowed the inclusion of adolescents with diverse clinical histories and treatment experiences. Epilepsy diagnosis was self-reported by participants, who confirmed having received an official clinical diagnosis from a certified neurologist or physician. A total of approximately 1552 eligible adolescents were approached during routine clinical visits or via patient association networks. Of these, 333 declined to participate or did not return completed questionnaires. This yielded a final analytical sample of 1219 adolescents (response rate of 78.54%). Inclusion criteria were defined as follows: (a) confirmed diagnosis of epilepsy for at least 6 months, ensuring sufficient exposure to treatment management; (b) age between 13 and 18 years, corresponding to a developmental period in which increasing autonomy in health-related decision-making typically emerges; (c) current engagement in a prescribed treatment regimen, including anti-epileptic medication; (d) sufficient proficiency in Italian to complete self-report measures independently. Detailed sociodemographic and clinical characteristics of the sample, including duration of illness, seizure frequency, seizure type, prescribed anti-epileptic medications, and etiology, are presented in Table 1. The target sample size was evaluated prior to data collection using an a priori power analysis for structural equation and mediation models. To detect small-to-medium direct and indirect path coefficients with statistical power set at 0.80 and an alpha level of α = 0.05, a minimum sample of approximately 400 participants was required. Our final sample of 1219 participants substantially exceeds this requirement, ensuring robust statistical power to evaluate the hypothesized structural mediation model.

### 2.2. Procedures

Data collection was conducted in a single assessment session in which participants completed a structured set of self-report questionnaires assessing psychological, behavioral, and contextual variables relevant to the study. Participants completed the survey battery digitally via an online platform. Completion time ranged between 15 and 20 min. Ethical approval was granted by the Institutional Review Board of the Institute for the Study of Psychotherapy on 26 February 2024 (ISP-IRB-2024–2). The study was conducted in full accordance with the Declaration of Helsinki and national ethical guidelines. Written informed consent was obtained from parents or legal guardians, alongside written assent from all adolescent participants. Participation was entirely voluntary, with explicit assurance that withdrawal was permitted at any point without impacting medical care. To ensure confidentiality, all data were collected anonymously, fully de-identified prior to analysis, and managed in compliance with applicable data protection regulations.

### 2.3. Measures

All constructs were evaluated using established self-report instruments. Specifically, formally validated Italian adaptations were utilized for life satisfaction and medication adherence, whereas the Italian version used in other studies was administered for autonomy-supportive healthcare (Graffigna et al., 2017).

Medication adherence was assessed using the Medication Adherence Report Scale (MARS), a 5-item self-report instrument specifically designed to evaluate adherence behaviors related to the use of medication (Tommelein et al., 2014). Responses are given on a 5-point Likert scale ranging from 1 (always) to 5 (never). Higher scores indicate better adherence. The scale showed satisfactory internal consistency in the present study (α = 0.83). Several studies confirm the psychometric properties of the scale in Italian and adolescent samples (Kosse et al., 2019; Liquori et al., 2022).

Life satisfaction was measured using the Satisfaction With Life Scale (SWLS), a 5-item instrument assessing global cognitive evaluations of one’s life (Diener et al., 1985). Responses are rated on a 7-point Likert scale from 1 (strongly disagree) to 7 (strongly agree). Higher scores reflect greater life satisfaction. Internal consistency was high in the present sample (α = 0.91). The SWLS demonstrated good psychometric properties in Italian adolescents (Di Fabio & Gori, 2016).

ASH was assessed using the Health Care Climate Questionnaire (HCCQ), a 6-item scale that measures perceived autonomy support from healthcare providers (Williams et al., 1996). Items assess the extent to which adolescents feel understood, listened to, and supported in treatment decisions. Responses are rated on a 7-point Likert scale (α = 0.92). Several studies confirm the psychometric properties of the scale in Italian and adolescent samples (Goethals et al., 2020; Graffigna et al., 2017).

### 2.4. Data Analysis

Data analyses were conducted using IBM SPSS Statistics Version 32.0.0 and R (lavaan package) Version 2026.06.0+242.pro13. Prior to hypothesis testing, descriptive statistics (means, standard deviations, skewness, and kurtosis) were computed to examine data distribution and suitability for parametric analyses. Data collection was conducted digitally using a forced-response layout to prevent unintentional item omission. To respect participant autonomy and to uphold ethical standards, “I prefer not to answer” options were included for all items. These response choices were treated as user-defined missing data (<1% across all items) and handled using Full Information Maximum Likelihood (FIML) estimation in lavaan. Model estimation used the Maximum Likelihood (ML) estimator.

To evaluate potential common method variance, Harman’s single-factor test was conducted as an exploratory diagnostic procedure.

Measurement validity was assessed through confirmatory factor analyses (CFAs) to evaluate the fit of the measurement models underlying the latent constructs. Model estimation used the Maximum Likelihood estimator in R (lavaan package) Version 2026.06.0+242.pro13. Missing values were handled via (FIML). Model fit was examined using multiple indices, including CFI, TLI, RMSEA, and SRMR. Because RMSEA is known to be systematically inflated in brief unidimensional measurement models with low degrees of freedom (Kenny et al., 2015), overall measurement model adequacy was evaluated based on the pattern of all fit indices combined, even if some RMSEA values were slightly above the optimal values.

Pearson correlation analyses were conducted to examine associations among ASH, medication adherence, and life satisfaction.

To test mediation hypotheses, structural equation modeling (SEM) with latent variables was employed. Consistent with the theoretical focus of the study, ASH was specified as the focal predictor, with medication adherence modeled as a potential mediator between ASH and life satisfaction. To preserve statistical power and improve model parsimony, item parceling was implemented for the latent variables in the structural model using a random approach, creating three parcels per construct (factor loadings ranging from 0.72 to 0.93). Indirect effects were estimated using bootstrap resampling procedures with 5000 iterations, generating bias-corrected confidence intervals. Model estimation used maximum likelihood estimation, which provides robust parameter estimates under conditions of non-normality. No post-hoc model modifications (such as covariance between error terms or cross-loadings) were applied. Model fit was evaluated using standard thresholds for goodness-of-fit indices (CFI ≥ 0.95, RMSEA ≤ 0.08, SRMR ≤ 0.08).

To examine whether clinical and sociodemographic variables influenced the structural relationships, a sensitivity analysis was conducted by re-running the mediation SEM including age, sex, epilepsy duration, monthly seizure frequency, medication burden (number of prescribed medications), and parental education as covariates on all study variables. The inclusion of these covariates did not significantly alter the primary structural paths or the indirect effect of ASH on life satisfaction via medication adherence (*β* = 0.14, *p* < 0.001). Furthermore, overall model fit remained robust: χ^2^(60) = 93.35, *p* = 0.004; χ^2^/df = 1.56; CFI = 0.994; RMSEA = 0.021 (90% CI [0.012, 0.029]); SRMR = 0.020. For clarity and parsimony, the unadjusted theoretical baseline model is retained in the main text.

## 3. Results

### 3.1. Preliminary Analyses

Table 1 reports the sociodemographic and clinical characteristics of the sample. The distribution of sex was balanced (49.1% boys, 50.9% girls). When considering gender identity, the majority identified as cisgender (42.6% boys, 47.8% girls), while smaller proportions identified as transgender boys (4.1%), transgender girls (2.5%), or non-binary (3.0%). Most participants identified as White (87.1%), with smaller proportions identifying as Black (4.6%), Latinx (2.3%), Asian (1.8%), or multi-ethnic (4.2%). Most adolescents were single (75.6%). Parental education levels were distributed across categories, with high school diploma representing the most frequent level for both mothers (46.4%) and fathers (49.1%). Clinical characteristics showed variability in epilepsy duration, seizure frequency, seizure type, medication load, and etiology, reflecting a heterogeneous clinical sample. Notably, 41.3% reported no seizures in the past month, while the majority of remaining participants reported low-to-moderate seizure frequency. Most cases were idiopathic (57.2%).

Table 2 presents descriptive statistics for all study variables. ASH (*M* = 5.30, *SD* = 1.60) indicated moderate-to-high levels of autonomy support. Medication adherence was above the scale midpoint (*M* = 3.23, *SD* = 0.90), and life satisfaction was moderate-to-high (*M* = 4.55, *SD* = 1.62). Skewness and kurtosis values indicated acceptable distributional properties for all variables.

A Harman’s single-factor test indicated that the first factor accounted for 12.55% of the total variance. While this suggests that common method variance is unlikely to be a primary driver of the observed relationships, this diagnostic test alone cannot fully rule out common method bias given the self-report methodology.

Confirmatory factor analyses supported the adequacy of the measurement models (Table 3). All constructs demonstrated acceptable-to-excellent fit across indices (CFI, RMSEA, SRMR, and TLI), indicating that the hypothesized factor structures adequately represented the observed data. Although RMSEA values for individual brief measurement models were elevated, potentially due to low degrees of freedom (Kenny et al., 2015), all the other values indicate satisfactory overall measurement model adequacy.

### 3.2. Correlational Analyses

Pearson correlations are reported in Table 4. ASH showed moderate positive associations with medication adherence (*r* = 0.36, *p* < 0.001) and life satisfaction (*r* = 0.36, *p* < 0.001). Medication adherence was also moderately associated with life satisfaction (*r* = 0.39, *p* < 0.001), indicating a robust relationship between treatment behavior and subjective well-being.

### 3.3. Mediation Analysis

**Figure 1:**
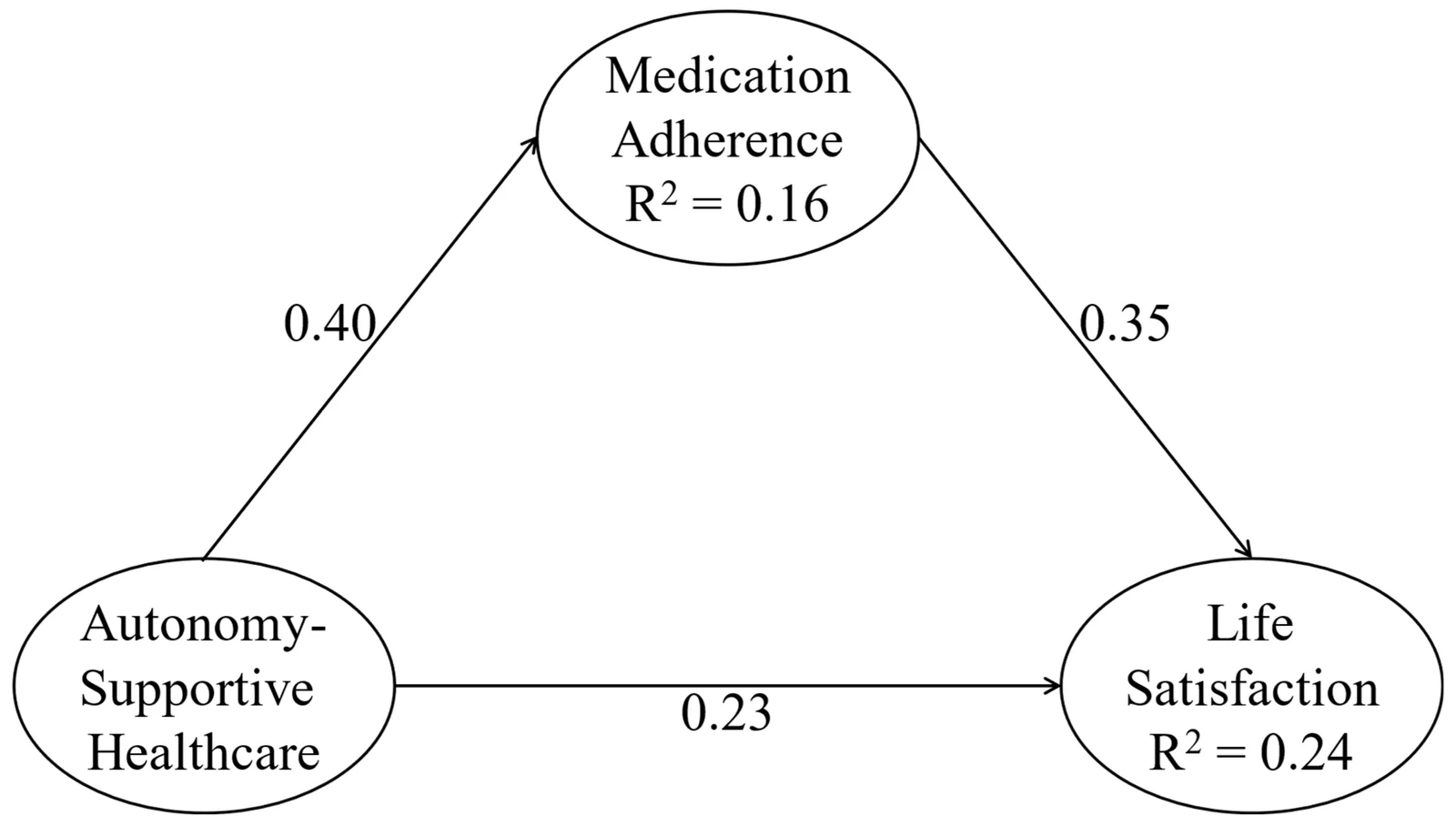
Structural Model. Note: Only direct paths are reported for presentation and clarity purposes; parcels were not presented for presentation and clarity purposes.

The hypothesized mediation model (see Figure 1) demonstrated good fit to the data: χ^2^(24) = 58.69, *p* < 0.001; χ^2^/df = 2.45; CFI = 0.994; RMSEA = 0.034 (90% CI [0.023, 0.046]); SRMR = 0.029. ASH was significantly associated with medication adherence (*β* = 0.40, *p* < 0.001) and life satisfaction (*β* = 0.23, *p* < 0.001). Medication adherence was positively associated with life satisfaction (*β* = 0.35, *p* < 0.001). Importantly, medication adherence significantly mediated the association between ASH and life satisfaction (*β* = 0.08, *p* < 0.001), as confirmed by bootstrap confidence intervals (see Table 5).

## 4. Discussion

This study examined the cross-sectional associations between perceived ASH, medication adherence, and life satisfaction in adolescents with epilepsy, grounded in SDT. Overall, the findings underscore patterns of co-occurrence between perceived autonomy-supportive healthcare environments, treatment-related behaviors, and psychological well-being during adolescence. A first key finding is the positive association between ASH and medication adherence. Adolescents who perceived their healthcare providers as more autonomy-supportive reported higher levels of adherence to prescribed treatment. This aligns with SDT frameworks, which theorize that autonomy-supportive contexts may be linked to greater internalization of health behaviors and self-regulation (Chen et al., 2019). These results are consistent with previous research showing that perceived support from healthcare professionals correlates with better treatment engagement in chronic illness populations (Barello et al., 2020; Cho et al., 2023). In adolescence, a developmental period characterized by increasing autonomy and identity formation (Branje et al., 2021; Calaresi et al., 2025; Saladino et al., 2025), supportive healthcare relationships may be observably relevant to adherence behaviors (Donahue et al., 2025). A second finding is the association between ASH and life satisfaction. Adolescents who perceived their healthcare environment as more autonomy-supportive reported higher global well-being. Prior research suggests that autonomy-supportive interpersonal contexts are positively related to psychological functioning, potentially through the satisfaction of basic psychological needs (Kaya et al., 2020; Tang et al., 2020). Consistently, adolescents who feel respected, heard, and involved in decision-making processes tend to report more positive psychosocial adjustment (Jordan et al., 2018; Nogg et al., 2021; Verrastro et al., 2024). In this sense, positive healthcare interactions may co-occur with broader evaluations of quality of life (Kang, 2023). A third key result is the positive association between medication adherence and life satisfaction. Adolescents with higher adherence reported greater well-being, suggesting that consistent engagement in treatment may correspond to a sense of health stability, structure, and perceived control. These findings are in line with previous evidence linking adherence in chronic illness contexts to better emotional and psychosocial outcomes in youth (Byansi et al., 2023; Castro & Brito, 2023). From a broader psychological perspective, self-management behaviors may share a positive relationship with feelings of competence and agency during adolescence. The mediation analysis suggested that medication adherence partially explained the association between ASH and life satisfaction. Of note, because these findings are based on cross-sectional data, temporal ordering and causal directionality cannot be established; thus, the observed mediation effect is best understood as a cross-sectional indirect association rather than a causal sequence or definitive mechanism. Indeed, these results demonstrate observational associations rather than evidence that autonomy-supportive interventions directly improve adherence or well-being. Specifically, adolescents perceiving greater autonomy support from healthcare providers reported higher adherence, which in turn was associated with greater life satisfaction. This pattern is consistent with SDT-based models in which supportive contextual factors relate indirectly to well-being through health behavior regulation (Eassey et al., 2020; Raaijmakers et al., 2014). These results indicate that the association between autonomy-supportive healthcare and adolescents’ well-being may operate, at least in part, through more consistent engagement in prescribed treatment (Paternoster et al., 2025). Rather than functioning solely as a behavioral outcome, medication adherence may represent a proximal link between healthcare context and broader indicators of psychological functioning and perceived quality of life.

Furthermore, although clinical factors such as seizure frequency, epilepsy duration, and medication burden represent important aspects of the disease experience, our sensitivity analyses demonstrated that the core associations between autonomy-supportive healthcare, medication adherence, and life satisfaction remained robust even when controlling for these variables. This suggests that the psychological and relational mechanisms central to Self-Determination Theory may operate relatively independently of objective clinical severity. Nevertheless, future research should explore whether specific clinical subgroups (e.g., individuals with drug-resistant epilepsy vs. those in full remission) experience distinct relational dynamics in healthcare settings.

Some alternative explanations and methodological considerations must be noted when interpreting these findings. First, although our sensitivity analysis demonstrated that controlling for key sociodemographic and clinical covariates (e.g., age, sex, illness duration, seizure frequency, medication burden, and parental education) did not significantly alter the primary structural paths or the indirect effect, the potential impact of unmeasured confounding cannot be fully ruled out. Variables such as family functionality, parental autonomy support, comorbid psychiatric conditions (e.g., anxiety or depression), and cognitive/executive functioning were not included in the model and could independently influence both adherence and global life satisfaction. Second, alternative conceptual mechanisms may account for the observed patterns. For example, adolescents with higher baseline psychological well-being or greater emotional resilience may perceive their healthcare providers more favorably (a reverse perception bias) and demonstrate superior self-management capacity. Similarly, higher medication adherence may not directly enhance life satisfaction but rather serve as a marker for broader conscientiousness or systemic family support. Future longitudinal and experimental designs are necessary to isolate these pathways, control for unmeasured psychiatric and familial confounders, and establish true directional mechanisms. Furthermore, while our conceptual model was grounded in Self-Determination Theory, the cross-sectional nature of this study precludes definitive conclusions regarding the temporal sequence of these constructs. Alternative directional pathways remain equally plausible. For instance, adolescents with higher baseline life satisfaction and greater psychological well-being may possess more self-regulatory resources, leading to higher medication adherence. Similarly, well-adjusted individuals may evaluate their clinical interactions more favorably, perceiving greater autonomy support from their healthcare providers. Prospective longitudinal designs or experimental intervention studies are necessary to disentangle these reciprocal dynamics and determine the precise directional mechanisms over time.

This study possesses several notable strengths that contribute to the literature on pediatric epilepsy self-management. First, the investigation utilizes an unusually large, multi-site sample of adolescents with epilepsy recruited across Northern, Central, and Southern Italy, enhancing both statistical power and geographical generalizability. Second, the integration of Self-Determination Theory provides a robust, theoretically driven framework for examining the indirect mechanisms linking healthcare climate to subjective well-being. Third, from a methodological standpoint, the study employed rigorous analytical procedures, including Structural Equation Modeling with latent variables to account for measurement error, FIML to handle missing data without sample loss, and robust sensitivity analyses adjusting for key sociodemographic and clinical covariates.

### 4.1. Limitations and Future Directions

Several limitations should be acknowledged. First, the reliance on self-report measures may introduce bias related to social desirability and recall inaccuracies. Adolescents may overreport adaptive behaviors such as medication adherence or underreport difficulties, which could relate to the observed associations. Similarly, while a Harman’s single-factor test indicated that the first factor accounted for only 12.55% of the total variance, it must be acknowledged that this diagnostic technique alone cannot fully rule out common method variance or social desirability bias. Second, the cross-sectional design precludes causal inference. Although findings are consistent with theoretical expectations derived from Self-Determination Theory, temporal ordering of effects cannot be established. Third, the model focused on perceived autonomy-supportive healthcare and did not fully account for clinical or contextual factors (e.g., illness severity, comorbidities, family support) that may also shape adherence and well-being.

Future research should employ longitudinal designs to clarify the directionality and stability of the observed associations. Furthermore, testing alternative structural models (such as reciprocal or reversed directional paths) across multi-wave data would strengthen the structural analysis and further elucidate these complex behavioral and psychological pathways. Similarly, the inclusion of objective or multi-informant measures of adherence would help reduce shared method variance. In addition, integrating clinical and relational contextual factors would allow for a more comprehensive understanding of how autonomy-supportive healthcare processes operate within broader systems relating to adolescent adjustment in chronic illness. Future research should also investigate autonomy-supportive healthcare using routinely collected clinical documentation rather than relying exclusively on self-report measures. Because these relational aspects of care are frequently recorded as heterogeneous free-text narratives, natural language processing approaches may represent an important step toward their standardization and large-scale evaluation in real-world clinical settings (Vanalli et al., 2023).

### 4.2. Practical and Clinical Implications

While these cross-sectional findings cannot establish whether adopting specific communication techniques will directly enhance clinical outcomes, they highlight the observational relevance of relational aspects of care alongside medical management. Pending longitudinal or interventional validation, clinicians might consider communication styles that align with patient autonomy, such as providing clear rationales for treatment, encouraging participation in decisions, and acknowledging patients’ perspectives, as potential avenues to foster patient engagement. In adolescence, when autonomy and identity development are particularly salient, exploring such communication practices may be a helpful observational framework for care teams. These observational patterns also highlight the potential utility of multidisciplinary care models in addressing psychosocial barriers to adherence, while supporting family involvement in a developmentally sensitive manner. To translate these findings into everyday practice, clinicians must understand what autonomy-supportive healthcare (ASH) looks like in routine pediatric epilepsy care. Rather than taking a purely paternalistic approach (e.g., simply dictating a medication schedule), an ASH environment involves engaging adolescents as active partners in their management. In practice, this includes:

Providing rationale: Explaining why daily anti-seizure medication (ASM) is necessary in relatable, age-appropriate terms (e.g., connecting adherence directly to their personal goals, such as maintaining a driving license, participating in sports, or staying independent from parental overprotection).

Offering meaningful choices: Involving the adolescent in decisions regarding dosage schedules or formulations when clinically feasible (e.g., choosing once-daily vs. twice-daily dosing to fit school routines).

Non-judgmental communication: Acknowledging the frustration and side effects associated with chronic ASM use without using shaming or punitive language when non-adherence occurs.

Realistically, when clinicians foster this environment, adolescents feel their agency is respected rather than threatened. This shift internalizes their motivation to manage their illness, making daily medication adherence feel less like forced compliance and more like a self-determined behavior that protects their autonomy and quality of life.

Translating autonomy-supportive healthcare into practice is especially critical during the transition from pediatric to adult epilepsy services, a vulnerable developmental window for adolescents. Recent qualitative evidence from Italian epilepsy centers reveals a widespread lack of standardized transition protocols, with complex patients facing significant barriers to multidisciplinary care (Viganò et al., 2026). Integrating autonomy-supportive principles (e.g., structured joint consultations, encouraging direct patient communication, and gradual self-management transfer) into standardized national transition pathways could provide the practical framework needed to sustain treatment adherence and life satisfaction as youth move into adult care systems.

Furthermore, the impact of autonomy-supportive healthcare may not be uniform across all adolescents with epilepsy, but rather contingent upon individual care and nursing complexity, as well as broader sociocultural factors. The recent literature in the Italian context emphasizes that patient complexity encompasses both medical burden and nursing care intensity. Adolescents with high care complexity, such as those with severe drug-resistant epilepsy, neurological comorbidities, or high daily nursing needs, may experience different relational dynamics than those with lower care requirements. For highly complex patients, autonomy support must be carefully balanced with structured clinical guidance and direct nursing assistance, as overemphasizing independent decision-making without adequate functional support could inadvertently create burden. Additionally, epilepsy-related perceptions, knowledge, and stigma vary across Italian regions and stakeholder groups (Romozzi et al., 2026). This underscores that healthcare environments do not exist in isolation; fostering autonomy-supportive care requires acknowledging patient complexity, addressing regional disparities, and combatting public stigma to create a supportive continuum of care.

## 5. Conclusions

Overall, this study provides evidence that perceived autonomy-supportive healthcare is cross-sectionally associated with both medication adherence and life satisfaction in adolescents with epilepsy. To our knowledge, this study represents one of the first large-scale empirical investigations to demonstrate the utility of Self-Determination Theory in explaining the interplay between healthcare climate, medication adherence, and global life satisfaction in a nationwide cohort of youth with epilepsy. These findings suggest that the relational quality of healthcare interactions co-occurs with both treatment engagement and psychological well-being during adolescence. Although the cross-sectional design limits causal interpretation, the observed associations are consistent with an indirect association in which supportive healthcare environments relate to higher life satisfaction in part alongside more consistent medication adherence. Rather than establishing a directional pathway, this study highlights medication adherence as a correlate-based behavioral link that warrants further prospective investigation. Taken together, these results underscore the importance of patient-centered, autonomy-supportive approaches in adolescent epilepsy care and support the need for longitudinal research to better clarify how healthcare experiences translate into long-term behavioral and psychological outcomes.

## Figures and Tables

**Table 1:** Sociodemographic and Clinical Characteristics of the Participants.

Variable	%
Sex	
Boys	49.1
Girls	50.9
Gender Identity	
Cisgender Boys	42.6
Cisgender Girls	47.8
Transgender Boys	4.1
Transgender Girls	2.5
Non-Binary	3.0
Ethnicity Identity	
Asian	1.8
Black	4.6
Latinx	2.3
Multi-ethnic	4.2
White	87.1
Relationship Status	
Single	75.6
In a relationship	24.4
Maternal Education	
Elementary School	13.5
Middle School	28.1
High School Diploma	46.4
Bachelor’s Degree	8.4
Postgraduate Degree	3.6
Paternal Education	
Elementary School	13.1
Middle School	24.6
High School Diploma	49.1
Bachelor’s Degree	11.2
Postgraduate Degree	2.0
Years of Epilepsy	
1–2	14.3
3–4	20.2
5–6	18.7
7–8	22.7
9–10 or more	24.1
Monthly Seizures	
0	41.3
1–5	44.9
6–10	7.4
11–15 or more	6.4
Type of Seizures	
Partial	53.2
Generalized	46.8
Prescribed Medications	
1	45.8
2	29.1
3 or more	25.1
Cause of Epilepsy	
Idiopathic	57.2
Symptomatic	42.8

**Table 2:** Descriptive Statistics of the Study Variables.

Variables	*Min*	*Max*	*M*	*SD*	*Sk*	*K*	α
1. ASH	1	7	5.30	1.60	−0.99	0.36	0.92
2. Medication Adherence	1	5	3.23	0.90	−0.37	−0.45	0.83
3. Life Satisfaction	1	7	4.55	1.62	−0.15	0.05	0.91

Note: ASH = autonomy-supportive healthcare.

**Table 3:** Goodness-of-Fit Indices of the Measurement Models.

Variable	df	GFI	AGFI	NFI	CFI	RMSEA	SRMR	TLI
ASH	9	0.969	0.919	0.982	0.983	0.104	0.035	0.969
Medication Adherence	5	0.981	0.942	0.973	0.976	0.093	0.028	0.951
Life Satisfaction	5	0.990	0.969	0.993	0.994	0.066	0.012	0.988

Note: ASH = autonomy-supportive healthcare. Factor loadings ranged from 0.61 to 0.84.

**Table 4:** Pearson’s Correlations.

Variable	1	2
1. ASH	-	
2. Medication Adherence	0.36 ***	-
3. Life Satisfaction	0.36 ***	0.39 ***

Note: *** *p* < 0.001. ASH = autonomy-supportive healthcare.

**Table 5:** Path Estimates, Standard Errors and 95% Confidence Intervals of the Indirect Effects.

	*β*	*B*	*p*	SE	CI	CI
					*LL*	*UL*
**Direct Effect**						
ASH → Medication Adherence	0.40	0.22	<0.001	0.02	0.18	0.26
ASH → Life Satisfaction	0.23	0.23	<0.001	0.04	0.15	0.30
Medication Adherence → Life Satisfaction	0.35	0.63	<0.001	0.07	0.50	0.76
**Indirect Effect via Medication Adherence**						
ASH → Life Satisfaction	0.08	0.14	<0.001	0.02	0.10	0.18

Note: *β* = standardized beta values; *B* = unstandardized beta values. The reported SE and CI values were computed using 5000 bias-corrected bootstrap resamples and refer exclusively to the unstandardized (B) metric. ASH = autonomy-supportive healthcare.

## Data Availability

The data presented in this study are available on request from the corresponding author.
